# Effect of Test-Based versus Presumptive Treatment of Malaria in Under-Five Children in Rural Ghana – A Cluster-Randomised Trial

**DOI:** 10.1371/journal.pone.0152960

**Published:** 2016-04-07

**Authors:** Frank Baiden, Jane Bruce, Jayne Webster, Mathilda Tivura, Rupert Delmini, Seeba Amengo-Etego, Seth Owusu-Agyei, Daniel Chandramohan

**Affiliations:** 1 Epidemiology Unit, Ensign College of Public Health, Kpong, Eastern Region, Ghana; 2 Department of Disease Control, London School of Hygiene and Tropical Medicine, London WC1E 7HT, United Kingdom; 3 ACT Trial Team, Kintampo Health Research Centre, Kintampo, Brong Ahafo Region, Ghana; University of California Los Angeles, UNITED STATES

## Abstract

**Background:**

Malaria-endemic countries in sub-Saharan Africa are shifting from the presumptive approach that is based on clinical judgement (CJ) to the test-based approach that is based on confirmation through test with rapid diagnostic tests (RDT). It has been suggested that the loss of the prophylactic effect of presumptive-administered ACT in children who do not have malaria will result in increase in their risk of malaria and anaemia.

**Methods and Findings:**

We undertook a cluster-randomized controlled trial to compare the effects of the presumptive approach using clinical judgment (CJ-arm) and the test-based approach using RDTs (RDT-arm in a high-transmission setting in Ghana. A total of 3046 eligible children (1527 in the RDT arm and 1519 in the CJ- arm) living around 32 health centres were enrolled. Nearly half were female (48.7%) and 47.8% were below the age of 12 months as at enrolment. Over 24-months, the incidence of all episodes of malaria following the first febrile illness was 0.64 (95% CI 0.49–0.82) and 0.76 (0.63–0.93) per child per year in the RDT and CJ arms respectively (adjusted rate ratio 1.13 (0.82–1.55). After the first episode of febrile illness, the incidence of severe anaemia was the same in both arms (0.11 per child per year) and that of moderate anaemia was 0.16 (0.13–0.21) vs. 0.17 (0.14–0.21) per child year respectively. The incidence of severe febrile illness was 0.15 (0.09, 0.24) in the RDT arm compared to 0.17 (0.11, 0.28) per child per year respectively. The proportion of fever cases receiving ACT was lower in the RDT arm (72% vs 81%; p = 0.02).

**Conclusion:**

The test-based approach to the management of malaria did not increase the incidence of malaria or anaemia among under-five children in this setting.

**Trial Registration:**

ClinicalTrials.gov NCT00832754

## Introduction

Although appreciable progress has been made in the control of malaria the disease remains an important cause of morbidity and mortality in endemic countries in sub-Saharan Africa.[1,2] Diagnosis has been a major challenge confronting effective clinical management of malaria. The approach to malaria diagnosis has important implications for its effective prevention and control. [3,4]

The two main approaches to the diagnosis of malaria are the presumptive and test-based approaches. The presumptive (or clinical judgement-based) approach relies solely on presenting signs and symptoms to establish diagnosis while the test-based approach relies on parasitological confirmation. For many years, the presumptive approach has been used across all age groups and transmission settings. The approach was justified on the basis of the following: (1) A high proportion of febrile illnesses in high-endemic settings was due to malaria, and the disease took a rapidly-deteriorating course in under-five children. Treatment therefore needed to be initiated promptly. (2) Microscopy which was the only practical means for confirming the diagnosis of malaria was technically complex and not available in areas that had the highest burden of the disease. (3) The first-line drugs used to treat malaria before the introduction of artemisinin-combination treatment (chloroquine and sulphadoxine-pyrimethamine) were safe, effective and inexpensive.[5,6] The presumptive approach formed the basis of the management of malaria within the Integrated Management of Childhood Illnesses (IMCI). [7]

In early 2010, WHO issued revised guidelines for the management of malaria that called for a shift from the presumptive to the test-based approach, across all transmission settings and in all age groups. The justifications for the shift include: (1) Substantial decline in the levels of malaria transmission in many countries including some areas that were previously high-transmission settings; (2) Availability of reliable malaria rapid diagnostic tests (mRDTs) for use as a point-of-care device; (3) Artemisinin-based combination treatments (ACTs), the current drugs of choice for treatment of malaria, are relatively expensive.[8] Given the emergence of delayed parasite clearance with the use of ACT in endemic countries in Southeast Asia, there is an urgent need to reduce unwarranted use of ACTs, and the chances of resistance development.[9,10,11] The use of test to differentiate between malaria and non-malaria cases is also expected to lead to improved attention to the proper management of non-malarial febrile illnesses. It is also expected to lead to substantial reduction in ACT cost to national programmes.

Those who opposed the shift to this approach argued that reported reductions in the transmission of malaria were not widespread enough to justify a change in the treatment guidelines. They also hypothesized that reduction in ACT use will remove the protective effect of intermittent preventive treatment (IPT)[12] in children who test negative. This concern is based on the fact that in many settings in malaria-endemic sub-Saharan Africa, children aged less than 2 year suffer at least four episodes of febrile illnesses each year. About half of these episodes are non-malarial[13] and therefore restricting ACT to RDT positive cases alone could substantially reduce the number of children receiving ACT for a febrile episode. The preventive benefit of IPT has been demonstrated in studies across different transmission settings in sub-Saharan Africa[12,13,14] and the reduction in ACT use in this regard could imply a loss of the opportunistic prophylactic benefit it would offers.[15] This could lead to an increased risk of malaria in children who present with non-malarial febrile illnesses. The trial that demonstrated the efficacy of IPT used non-ACT medications and the protective effect, where established largely attributed to the non-artemisinin partner drug with a longer half-life.

Currently there are very few diagnostic tools that can be applied to determine the aetiology of non-malarial febrile illnesses at point-of-care, and on that basis decide when to prescribe antibiotics. In the absence of improved diagnostics tools for the management of these illnesses, test-based management of malaria could (conceptually-considered) also lead to substantial increase in the inappropriate use of antibiotics to manage such cases.

We report here on a cluster-randomized, controlled trial we undertook a comparison of the effects of test-based (intervention) versus presumptive (control) approaches to the management of malaria in rural Ghana. We hypothesized that test-based management of malaria will lead to reduction in ACT prescription and use. This will result in an increase in the incidence of malaria and anaemia, as well as in the prescription of antibiotics. The trial was registered in the online clinical trials registry as NCT00832754.

## Methods

The protocol for the study was approved by the Ethical Review Committees of KHRC, the Ghana Health Service (GHS), and the London School of Hygiene and Tropical Medicine (LSHTM). Signed (thumb printed if unable to read or write in English) informed consent was obtained from each caregiver prior to enrolment of the child. A Data Safety Monitoring Board (DSMB) periodically reviewed safety parameters and incidence of malaria and anaemia. The DSMB also approved the final analysis plan.

### Study area

The study was conducted in six districts in Ghana between February 2009 and July 2012. The districts lie within the forest-savannah transition zone which is considered to have perennially-high malaria transmission. According to data from routine health services, malaria is the leading cause of morbidity and mortality in the districts. In 2003–4, the Entomological Inoculation Rate (EIR) was estimated at 231–269 infective bites per person per year. [16] At the time of initiating the study health centres in the districts were using the presumptive approach in the management of fevers. The antimalarials used in the management of fevers were the nationally-approved first line ACTs: artesunate-amodiaquine, artemether-lumefantrine and dihydroartemisinin-piperaquine. In a cross-sectional study conducted during the course of the trial, the majority of caregivers preferred the test-based approach to the clinical judgement approach to managing fevers in under-five children. Caregivers considered the test-based approach to represent an improvement in health service delivery.[17]

### Selection and randomisation of health centres

The unit of randomisation was health centres. Thirty two health centres were stratified by districts, ranked by the number of Diptheria-Pertussis-Tetanus1 (DPT1) vaccinations given in the facility in the preceding year and paired according to the rank within each district. Within each pair of health facilities one was allocated randomly to implement test-based (RDT-arm) management of malaria, while the other health facility within the pair was allocated to implement the clinical judgement (CJ arm) approach. This was done using STATA software.

### Enrolment and data collection

Between January 2010 and May 2010, we enumerated all households that were located within 2km radius of the selected health centres and enrolled one hundred children per health facility who met the following criteria: (1) age <24 months; (2) caregiver indicated they used the index health centre as first point of call whenever the child was unwell; (3) caregiver was willing to give informed consent for the child to be enrolled in the study.

We enrolled children living closest to the selected health centres and extended outwards circumferentially till the target of 100 children was achieved. Enrolled children were given a study identification card with a unique identification code. We recruited thirty-two research fieldworkers and trained them on Good Clinical Practice (GCP) and the standard operating procedures of the study. We also trained them on how to take blood from finger prick to perform malaria RDTs, how to use of the Hemocue™ to measure haemoglobin levels, and how to prepare blood smears for microscopy. One fieldworker was stationed at each health centre. Each district had a research field supervisor who paid daily visits to each of the health centres to review work, collect completed study forms and blood samples.

All health workers in the participating facilities who took care of sick children were given refresher training in the management of febrile illnesses according to IMCI guidelines. This training was conducted just before commencement of the study and repeated every 4–6 months throughout the period of the study. Clinicians in all the participating health facilities were taken through the correct use and interpretation of RDT. This training relied on the findings of an earlier study into the accuracy of an HRP-2 RDT in the study area by the research team. In that study, a high rate of false-positive RDT results due to antigen persistence was observed.[18] We used this locally-generated evidence to teach clinicians to enquire about recent malaria treatment whenever they were confronted with positive RDT results. It was expected that clinicians from the control facilities would still find the knowledge useful to apply in the care of non-study patients. In the course of the trial any new health workers posted to a health centre were also taken through the protocol of the study and correct use of RDTs.

Each blood slide in the study was read independently by two expert microscopists at the reference laboratory at the Kintampo Health Research Centre (KHRC). Discrepancies were resolved through a third reading by a third microscopist.

### Intervention and control

In health facilities that were randomised to the test-based (intervention) approach, all enrolled children who reported with fever were tested with mRDT and received an ACT only when the test was positive. The mRDTs used in the study were procured from the Ghana National Malaria Control Program. Two brands of mRDTs procured through the Ghana National Malaria Control Programme, CareStart™ and First Response™, were used throughout the period of the study. They were also treated for any concurrent illnesses according to IMCI guidelines.

In health facilities that were randomised to the clinical judgement (control) approach, enrolled children who presented with fever were not tested for malaria. The treatment for malaria in these facilities was presumptive and based on presenting clinical symptoms and signs. The treatment of all other ailments were based on the IMCI guidelines.

The fieldworkers stationed in each (intervention and control) health facility helped to ensure compliance with the protocol by reminding attending clinicians about the protocol of the study, as applicable to the particular health centre. This was reinforced during the 4–6 monthly training sessions that were held for health workers in both intervention and control facilities.

### Surveillance for febrile illnesses

All caregivers of children enrolled into the study were advised to bring their sick children to the health centre. To overcome the possible effect of financial barrier, the project paid to have all the children enrolled under the Ghana National Health Insurance Scheme. Whenever a study child presented with axillary temperature of 37.5°C or above, or reported fever in the preceding 72 hours, the fieldworker took blood samples for blood smear microscopy and used the Hemocue® to determine haemoglobin level. The result of the haemoglobin test was made available to the attending health worker together with the result of mRDT if the health centre was an intervention facility. In facilities in the CJ arm, only haemoglobin test and blood smear microscopy were done. The results of the haemoglobin test were also made available to the attending health worker to guide clinical management.

As part of active surveillance, field supervisors visited study children at least once every two months and asked the caretakers about any febrile illnesses that may have occurred in the period in between visits. The field supervisors completed morbidity questionnaires during each visit. This process of surveillance was instituted to ensure that all febrile episodes that could have been reported to local pharmacies and chemical shops was captured. If a child was unwell at the time of the home visit or reported having had fever in the preceding 72 hours, the field supervisor issued a referral note and advised the caregiver to send the child to the study health centre.

### Sample size

The primary endpoint was incidence of malaria during the 24 months of follow-up after the first episode of febrile illness. It was estimated that to assess the effect of test-based management of malaria on the incidence of malaria in children under 48 months of age would require 30 clusters (15 per arm) and 200 person-years at risk (PYAR) per cluster. This was based on the following expectations: (1) Incidence of malaria in the CJ arm would be 0.8 episode/ 1 per child per year (the rate observed in a group of <24 month old children in Navrongo, Ghana [13]; (2) the coefficient of variation in the incidence of malaria between clusters will be 0.18; (3) The study would have 80% power at 95% significance level to detect a 20% difference in the incidence of malaria between the two comparison groups.

As the total number of children living around some of the selected health facilities was <100 an additional pair of health centres was selected randomly in order to reach the required number of children. Although mortality was measured in the study, it was assumed *a priori* that the number of deaths would not be sufficient to detect any significant difference between the two arms.

### Data management and analysis

Data were double-entered using FoxPro version 9. The analysis was conducted using STATA version 12. Intention-to-treat analysis was performed using all enrolled children while per protocol analysis was applied to children who adhered to correct assessment and treatment criteria. Person-time in the study for each child was calculated using dates on which children were seen at home visits or at a health facility. If a child was not seen for more than 3 months, the person-time was censored for this period until they were seen again. Malaria was defined as the presence of fever and a positive blood smear microscopy result. Fever was considered to be severe when it was associated with one of more of the IMCI danger signs of lethargy or drowsiness, vomiting everything, refusing all food including breastfeeding and convulsions.

The sociodemographic characteristics of children in the two arms of the enrolled cohort were measured and compared using Pearson’s chi-square corrected for clusters. Similarly fever episodes and corresponding blood test results and ACT prescriptions were compared. A multi-level Poisson model assuming unstructured covariance was used to estimate the incidence of malaria over the 24-month period to account for repeat episodes of malaria within a child, and clustering by heath facility. For the incidence of first or only episodes of malaria, moderate anaemia (haemoglobin between 5-8g/dL), severe anaemia (haemoglobin less than 5g/dL) and severe febrile illness, after the first febrile episode, a random effects Poisson model was applied, adjusting for age, socio economic status, and ITN use. In this report we present the survival analysis (in addition to the Poisson models) to show events over the entire follow-up period and also to offer opportunity for cross-reference with other studies.

## Results

**Fig 1:**
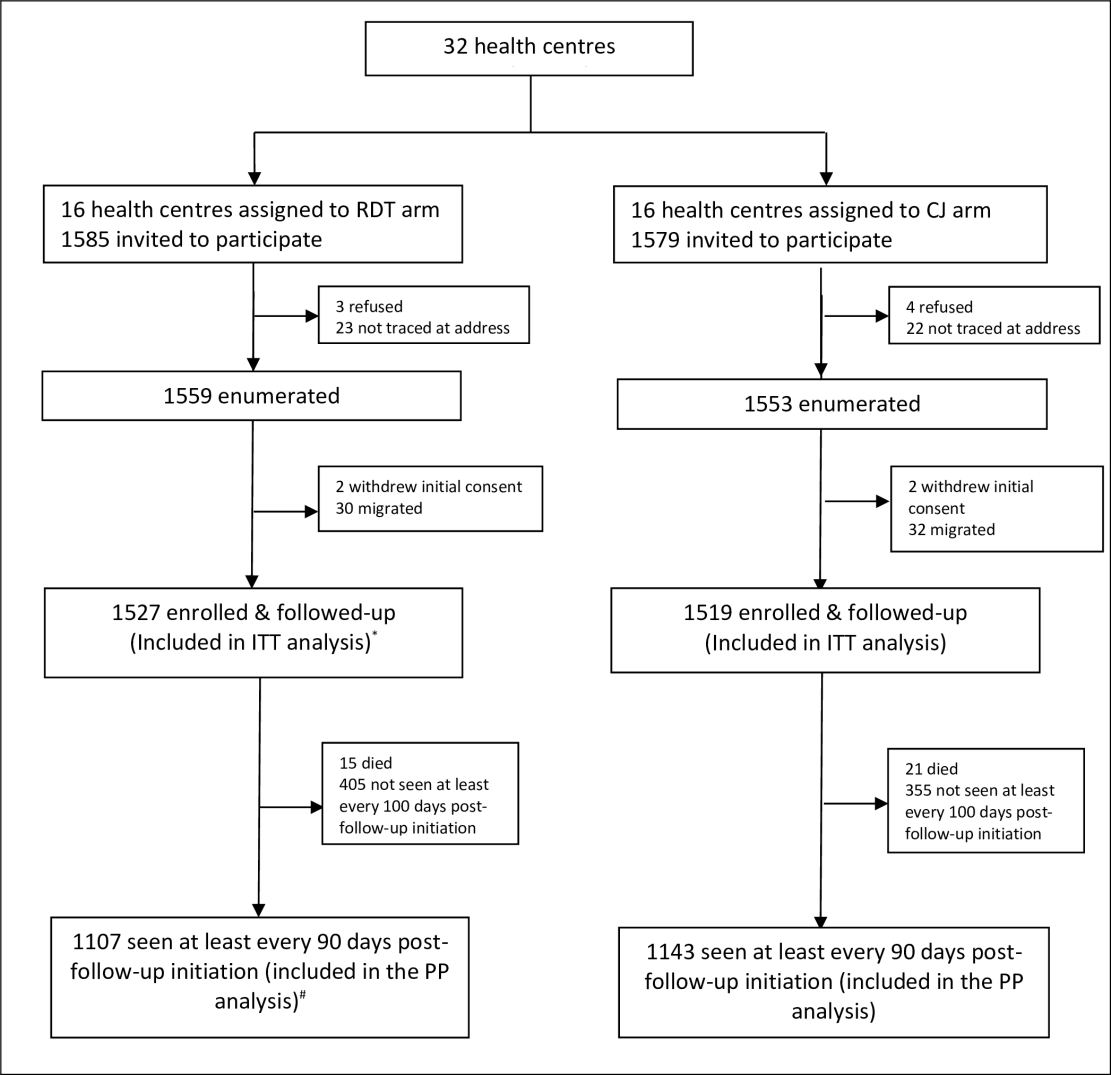
Trial Profile.

Potentially eligible children were enumerated between April 2010 and May 2010. Those who were available were consent and enrolled in June 2010 and followed up to June 2012. A total of 3046 eligible children (1527 in the RDT arm and 1519 in the CJ arm) were enrolled around the 32 health centres. (Fig 1) Nearly half of the study children were female (48.7%). Just about half 47.8% were also below the age of 12 months at the time of enrolment.

As part of active surveillance, 46,395 home visits were made over the 24-month follow-up period. The median number of home visits was 15 per child in both arms. 23.1% of children were referred to a health centre at least once following a home visit because they had fever or history of fever in the preceding 72 hours. The number of cases referred after homes visits were similar in both arms of the study (347 versus 357, p = 0.83). (**Table 1**) The mean number of passive health facility visits was 4.1 in the test-based arm and 4.3 in the CJ arm (p = 0.73). Two hundred and seventy five (9.0%) study children never visited the health centre throughout the follow-up period but they were regularly seen at home visits.

**Table 1:** Characteristics and comparability of children enrolled into the two arms of the study.

		Test-based approach (RDT)	Clinical judgement (CJ)	P-value
Total		1527*	1519*	
Age	< 12months	766 (50.2%)	691 (45.5%)	0.16
	≥12months	761 (49.8%)	828 (54.5%)	
Sex	Male	779 (51.1%)	782 (51.5%)	0.75
	Female	747 (49.0%)	736 (48.5%)	
Highest educational level of mother	None	425 (28.1%)	450 (29.7%)	0.75
	Primary	360 (23.7%)	341 (22.5%)	
	Secondary	715 (47.2%)	717 (47.3%)	
	Tertiary	15 (1.0%)	7 (0.5%)	
Highest educational level of father	None	345 (24.1%)	326 (22.0%)	0.72
	Primary	261 (18.2%)	267 (18.0%)	
	Secondary	752 (52.6%)	835 (56.2%)	
	Tertiary	70.0 (4.9%)	57 (3.8%)	
Ownership of bednet	Yes	1135 (74.4%)	1200 (79.0%)	0.26
	No	391 (25.6%)	319 (21.0%)	
Socioeconomic status	Most poor	311 (20.5%)	296 (19.5%)	0.67
	Poorer	267 (17.6%)	340 (22.4%)	
	Poor	290 (19.1%)	318 (21.0%)	
	Less poor	325 (21.4%)	278 (18.4%)	
	Least poor	324 (21.4%)	283 (18.7%)	
Immunization status	Incomplete	716 (52.3%)	704 (50.2%)	0.79
	Complete	653 (47.7%)	698 (49.8%)	
Number of home visits made	<15	458 (30.0%)	632 (41.6%)	0.46
	≥15	1069 (57.8%)	887 (58.4%)	
Weight on 1^st^ visit	<10kg	681 (49.9%)	641 (45.8%)	0.29
	≥10kg	684 (51.1%)	760 (54.3%)	
Time to 1^st^ fever	<131 days	608 (48.1%)	661 (51.6%)	0.39
	≥131 days	655 (51.9%)	620 (48.4%)	
Haemoglobin level at 1^st^ visit	<8gm/dl	365 (30.2%)	329 (28.0%)	0.39
	8-11gm/dl	711 (58.8%)	794 (58.3%)	
	>11gm/dl	133 (11.0%)	202 (13.7%)	

*Missing data in some variables account for numbers that are less than the total number of enrolled children.

The median time to first episode of fever was similar between the two arms (105 days in RDT versus 111 days in CJ arm). At their first visit to the health facility, 713 (29.1%) children had haemoglobin less than 8gm/dL. The median level of haemoglobin at the first contact was similar in both arms of the study (9.0 versus 9.2 gm/dL). The number of episodes of febrile illness per child over the 2 year study period was also similar between the two groups (3.7 vs 3.8 per child). In the RDT arm, 98.2% of febrile episodes seen at the health centres were tested with RDT and 72.3% were RDT positive. The sensitivity, specificity and positive predictive value for RDT use in the RDT-arm were 95%, 64% and 53% respectively. As a deviation from the study protocol, 8 out of 5799 (0.14%) cases that presented to CJ-arm facilities had RDT performed. (**Table 2**).

**Table 2:** RDT and blood smear result and ACT prescription.

	Test-based approach (RDT)		Clinical judgement (CJ)		P-value
	n	%	n	%	
**Total febrile episodes**	5673	49.5	5799	50.5	
Febrile episodes tested with RDT	5573	98.2	8	0.14	<0.001
Febrile Episodes receiving ACT	4100	72.3	4686	80.8	0.02
Febrile Episodes receiving antibiotics	3111	54.8	3260	56.2	0.78
RDT positive episodes	4112	73.8	4	50.0	0.06
RDT positive episodes receiving ACT	4023	97.8	4	100	0.80
RDT positive episodes receiving antibiotics	1813	44.1	2	50.0	0.75
RDT negative episodes	1461	26.2	4	50.0	0.06
RDT negative episodes receiving ACT	54	3.7	1	25.0	0.05
RDT negative episodes receiving antibiotics	1246	85.3	3	75.0	0.58
Blood Smear Positive episode	2795^*^	49.3	2914	50.3	0.85
Blood smear positive and RDT positive	2640	94.7	2	50.0	0.0001
Blood smear positive episode but did not receive ACT	181	6.5	360	12.4	0.04
Blood smear negative episodes that received ACT	1486	51.6	2132	73.9	<0.001
Blood smear negative episodes that received antibiotics	1926	66.9	1781	61.7	0.30
Blood smear positive episodes received antibiotics	1185	42.4	1479	50.8	0.16

^*^ RDT results were missing for 8 blood smear positive episodes.

### Intention-to-treat

#### Malaria

**Fig 2:**
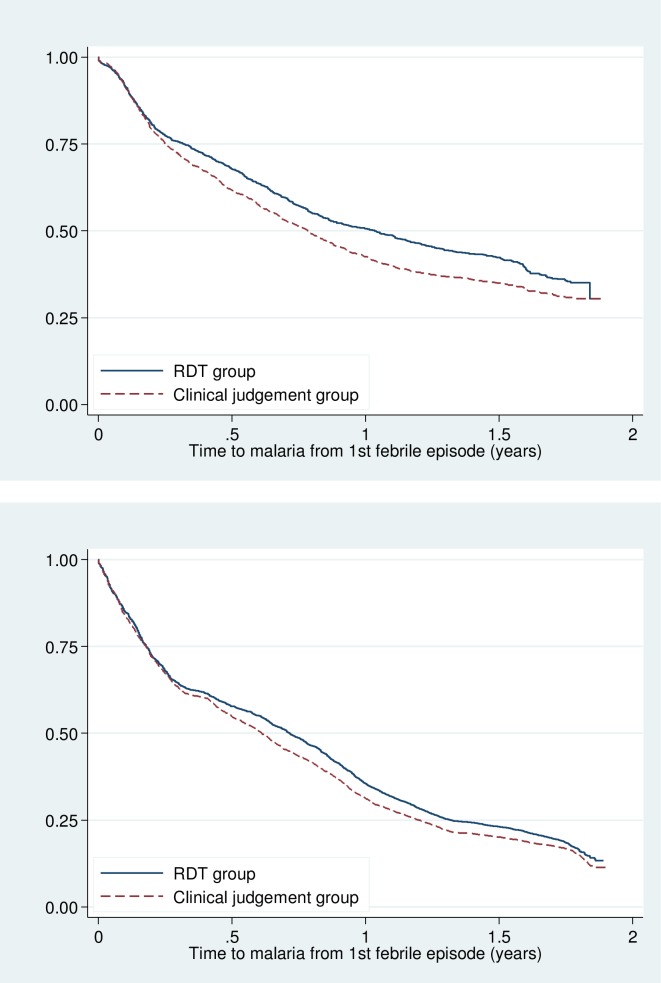
Survival curve of time to malaria after the first episode of a febrile illness in a) 1^st^ or only episode of malaria b) all episodes of malaria.

The incidence of all episodes (first or repeat) of malaria was 0.99 per child per year (95% CI 0.78, 1.27) in the RDT arm compared to 1.05 per child per year (95% CI 0.87, 1.29) in the CJ arm. There was no significant difference in the incidence of all episodes of malaria following the first episode of febrile illness (adjusted rate Ratio 1.13, 0.82–1.55) (Table 3). The survival time to malaria after the first episode of a febrile illness was similar for both the first or only episode of malaria and for all episodes of malaria (**Fig 2**). Within the RDT arm, the mean time to an episode of malaria following treatment with ACT for a febrile illness was 309 days compared to 252 days in those who did not receive an ACT for febrile illness, p = 0.009. The mean time to an episode of malaria for children who were RDT negative and therefore did not receive ACT but whose blood smear results were positive is 194 days.

**Table 3:** Incidence of health outcomes in RDT versus CJ arms.

		Test-based approach (RDT)		Clinical Judgement (CJ)		Rate Ratio (95%CI)	P-value
Number of children		1527		1519			
		Number of episodes	Incidence rate (95%CI)	Number of episodes	Incidence rate (95%CI)		
**Incidence of malaria**							
All episodes	Crude‡	2645	0.99 (0.78,1.27)	2806	1.05 (0.87,1.29)	1.09 (0.81,1.48)	0.53
	Adjusted†					1.09 (0.82,1.44)	0.57
After first episode of febrile illness	Crude‡	914	0.64 (0.49,0.82)	1011	0.76 (0.63,0.93)	1.12 (0.81,1.54)	0.50
	Adjusted†					1.13 (0.82,1.55)	0.47
Repeat malaria after first episode of febrile illness	Crude‡	697	0.35 (0.26,0.47)	778	0.41 (0.32,0.52)	1.08 (0.74,1.59)	0.68
	Adjusted†					1.09 (0.75,1.59)	0.64
**Incidence of severe febrile illness after first episode of febrile illness**	Crude‡	321	0.51 (0.09,0.24)	371	0.17 (0.11,0.28)	1.11 (0.56,2.15)	0.78
	Adjusted†					1.13 (0.58,2.21)	0.72
**Incidence of moderate after first episode of febrile illness**	Crude‡	349	0.16 (0.13,0.21)	358	0.17 (0.14,0.21)	1.02 (0.73,1.43)	0.91
	Adjusted†					1.04 (0.76,1.44)	0.80
**Incidence of severe anaemia after first episode of febrile illness**	Crude‡	266	0.11 (0.09,0.14)	279	0.11 (0.95,0.15)	1.05 (0.78,1.44)	0.74
	Adjusted†					1.04 (0.76,1.41)	0.82

‡ Crude estimates account for cluster-randomised design.

†Adjusted for cluster-randomised design and other covariates.

#### Severe febrile illness

A total of 1200 episodes of severe febrile illness occurred among 760 children during the period of follow-up. The incidence of severe febrile illness after a first episode was 0.15 (0.09–0.24) per child per year in the RDT arm compared with 0.17 (0.11–0.28) among children in the CJ arm (p = 0.78). The incidence of severe febrile illnesses was higher in children aged below 12 months of age at the time of enrolment (0.18 per child per year; 0.13–0.24) compared to those ≥12 months of age (0.14 per child per year; 0.10–0.21). (**Table 3**)

#### Anaemia

The incidence of moderate or severe anaemia in children following an episode of febrile illness was not significantly different between the two groups; 0.16 (0.13–0.21) vs. 0.17 (0.14–0.21) per child year and 0.11 (0.09–0.14) vs. 0.11 (0.95–0.15) per child year respectively. The adjusted rate ratios were 1.04 (0.76–1.44) and 1.04 (0.76–1.41) for moderate and severe anaemia respectively (**Table 3**).

#### Deaths

There were 21 (1.4%) deaths in the CJ arm compared with 15(1.0%) deaths in the RDT-arm. The difference was not statistically significant (p = 0.31).

#### ACT and antibiotic prescription

The proportion of fever cases that got treated with ACT was lower in the RDT arm compared to the CJ arm (72.3 vs. 80.8%; p = 0.02). Although the number of blood smears that were positive was similar in both arms (50.3% versus 53.3%; p = 0.61), the number of children who were blood smear positive but did not receive ACT was significantly higher in the CJ arm than in the RDT arm (12.5% versus 6.5%; p = 0.04) (**Table 2**).

The proportion of children receiving an antibiotic for a febrile episode was slightly lower in the RDT arm than in the CJ arm this difference was not statistically significant (48% vs 54%; p = 0.23). In the RDT arm the proportion of children receiving an antibiotic was higher among RDT negative compared to RDT positive children (85% vs. 44%; p < .0001). The proportion of children receiving a combination of an ACT and antibiotic was lower in the RDT arm but this was not statistically significant (32% for RDT arm vs. 41% in the CJ arm; p = 0.13). However the proportion of children who received only antibiotics was significantly higher in the CJ arm compared to the RDT arm (15.6% versus 22.9% p = 0.02). (**Table 3**)

### Per protocol analysis

The results of the per protocol analysis of all outcomes were similar to the intention to treat analysis (Data are not shown)

## Discussion

This study has used a cluster-randomised design to compare the effects of test-based management of malaria to the presumptive approach that had been practised for several decades. This study achieved a near-perfect provider adherence to test results or to clinical judgement. The observed incidence of 1.09 episodes per child per year in the CJ arm was higher than the assumed incidence of 0.8 episode per child year used in the sample size estimation. Thus the study was adequately powered to detect the anticipated 20% difference in the incidence of malaria. Given that adherence to the protocol was very good, the diagnosis of malaria was confirmed by microscopy and the study was adequately powered, we believe this study provides substantially robust evidence on the likely effects of test-based management of malaria in high transmission settings.

Previous studies that examined the effects of test-based management of malaria had been limited by the lack of appropriate comparator cohort[18,19,20], relatively smaller sample sizes[18], inadequate length of child follow-up [21], a failure to randomize the allocation of participants [18,19,20,21] and inadequate adherence to RDT-negative results. [22] These limitations should be considered when comparing the findings from those studies with the current study. There is however fair consistency across these studies to suggest that denying ACT to RDT-negative children does not lead to adverse treatment outcomes. In a study in two transmission settings in Tanzania involving 1000 children with a febrile illness, no significant adverse treatment outcomes were observed over a 7 day post-treatment follow up period among those children who were RDT negative and were not given an antimalarial drug. [19] Although it was demonstrated in a high-transmission setting in Ghana that the course of clinical recovery for RDT-negative children differed markedly from RDT-positive cases, overall treatment outcomes were comparable and no serious adverse effects were observed in children who were treated using the test-based approach.[18] Similar results were found in a low transmission setting in Kampala, Uganda where a representative sample of 601 children between one and ten years of age were followed up over a period of 18 months [21] and in north-eastern Tanzania where the test-based approach was adopted to treat 965 children enrolled over a period of one year.[20] In contrast a study in a high-transmission setting in Burkina Faso found that the effects of the test-based approach also depended on the ability of clinicians to identify and treat children with non-malarial febrile illnesses who are coincidentally test positive.[22] For the purpose of assessing the effects of test-based versus clinical judgement-based management of malaria however, none of the above-mentioned studies had an appropriate comparator arm and there was no random allocation to either test-based or clinical judgement-based treatment.

This main finding of this study is that mRDT-based treatment of febrile illness in <5 year old children did not result in an increase in the incidence of malaria, anaemia, severe febrile illnesses or the use of antibiotics in this high-transmission setting. These findings ran counter to our expectation that the test-based approach would deny a high proportion of children the prophylactic benefit that OPT offers in children whose fever is non-malarial.[15] The likely explanatory factors for this are the high background level of malaria transmission (blood smear positivity was 50.3% and 53.3% in RDT and CJ arms respectively) and the low RDT specificity (due to HRP-2 antigen persistence) which resulted in a high proportion of children in both arms being given ACT. The rather modest difference of 9% in the rate of ACT use between the two arms in this study is similar to the 14% observed in a similarly high-transmission setting in Burkina Faso.[22] It is however markedly lower than the 50% and 67% recorded in low-transmission settings in Zanzibar and north-eastern Tanzania respectively.[23,24]

Another factor that could explain the lack of significant effect on the risk of malaria was the significant difference in the proportion of children who were blood smear positive but did not receive ACT. This occurred more among children in the CJ-arm than among children in the RDT-arm (12.5% vs. 6.5%; p = 0.04). Given that previous studies have established that the strongest predictor of a repeat episode of malaria is an index episode of parasitaemia,[25,26] the higher false-negative diagnosis of parasitaemic children in the clinical judgement arm could have contributed to an increased risk of malaria in children in this group.

### Reduction in ACT use

An important factor that motivates the roll-out of test-based management of malaria is the expectation that it will lead to massive reduction in the wasteful use of ACTs.[5,6,24] The rather modest 9% reduction in ACT use observed in this study therefore raises concern about whether the expectation of massive reduction in ACT use could justify investments in the roll out of test-based management of malaria in high-transmission settings. This modest reduction was achieved in a rigorously-managed setting. In real-life settings where health worker adherence to RDT-negative results is unlikely to be optimal, the reduction in ACT use is likely to be even less. The contrast in extents of reduction in ACT use between high and low malaria transmission settings has been alluded to earlier. A cost-benefit analysis that included health worker adherence to test-results has also pointed to the fact that it might not be beneficial to implement test-based management of malaria in high-transmission settings (assuming reduction in ACT use is a major consideration) and the presumptive approach might be a more appropriate approach.[27] The expectation that test-based management of malaria will lead to massive reduction in ACT use cannot be strong enough basis to argue for its use.

### Quality of care and antibiotic use

An important finding in this study is the fact that compared with children in the RDT arm, a significantly higher number of smear positive children in the CJ arm did not receive ACT. This finding suggest that the presumptive approach is not an efficient means for reaching children who are parasitaemic and need to be given ACTs. Conversely the use of RDT significantly improves the targeting to ACTs to malaria cases. On this basis, the test-based approach can be said to lead to improvement in the quality of care.

In this study we found the use of HRP-2 RDT to have a PPV of 64%. This implies that possibly one in every three RDT positive cases could actually be blood smear negative and is RDT positive, likely on account of the persistence of HRP-2 antigens. This findings has implications for the training of clinicians on the interpretation of RDT results, the need for proper history-taking in the assessment of children and caution against over-reliance on RDT result to form the basis of the diagnosis of clinical malaria. There is also the need for caution in interpreting these findings to suggest a need for antibiotics in all cases where RDT result is positive but blood smear result is negative. In earlier work in the same study area, the positive yield on blood culture among RDT negative under-five children was less than 12%.[18]

A major concern about the shift to test-based approach has been the prospect that it could increase inappropriate use of antibiotics.^29-33^ The basis of the concern is that restricting ACT to confirmed malaria cases will increase the number of cases subsequently classified as non-malarial febrile illnesses, and in the absence of appropriate diagnostic tools these cases will be presumptively treated with antibiotics. In this study we found that prescription of antibiotics only or in combination with ACT was lower in the RDT arm compared to the CJ arm. However, the proportion of children treated with an antibiotic use was highest (85%) among children in the RDT arm who were RDT-negative. This compares with 44% among children in the RDT arm who tested positive, 51% and 62% among children in the CJ arm who (as later determined) were blood smear positive and negative respectively. The high antibiotic use in the RDT negative children did not however translate into overall significant increase in antibiotic use in the RDT arm (actually was reduced) because it was offset by reduction in use (44%) in children who had malaria and got confirmed with RDT. The relatively high background malaria prevalence implied that this reduction was large enough to offset the increased use in the RDT negative group. Our data suggests that the result of RDT (either positive or negative) will influence the decisions of health workers whether to use antibiotics or not. However, in high malaria transmission settings the reduction in antibiotic use in RDT-positive children would result in overall reduction in antibiotic use.

The pattern in antibiotic use and reduction in ACT use, without adverse effect on treatment outcomes suggest that test-based management of malaria improved the quality of care of both malaria and non-malaria febrile illnesses in this high transmission setting. The findings nevertheless add to available evidence on the need for further work to identify how best to target antibiotics use in the management of childhood febrile illnesses

The extrapolation of these findings to program settings is limited by a number of operational considerations. The level of provider adherence to test based management of malaria observed in this study was very high, good quality mRDT stored in optimum conditions, and the study children were closely monitored. Thus any deterioration in their clinical condition resulting from inadequate treatment was promptly identified and the caregivers brought the children for review as recommended. On the contrary, in routine health systems, the follow up of children is often not optimal, caregivers may not return for reviews, and the accuracy of mRDT may be variable.

## Conclusion

The application of test-based management of malaria did not increase the risk of malaria or anaemia in children in this setting. It improves the quality of care. It however did not lead to massive reduction in ACT use. The WHO recommendation of test-based management of malaria in children is justified in this setting.

## Supporting Information

S1 Checklist CONSORT 2010 Checklist-Completed. (DOC)

S1 Protocol The Protocol of the study. (DOC)
