# Supplementary material for: Effect of Test-Based versus Presumptive Treatment of Malaria in Under-Five Children in Rural Ghana – A Cluster-Randomised Trial
Source: PLoS One. 2016 Apr 7;11(4):e0152960. doi: 10.1371/journal.pone.0152960 (PMC4824463; doi:10.1371/journal.pone.0152960)
Supplement: S1 Protocol — (DOC) [file pone.0152960.s002.doc]

## ACT Consortium

***Project final proposals for external review and contracts 2008***

**TITLE OF PROPOSAL:**

Effects of restricting the use of Artesunate plus amodiaquine combination therapy to malaria cases confirmed by a dipstick test: A cluster randomised control trial

**PRINCIPAL INVESTIGATOR(S) AND AFFILIATIONS:**

Daniel Chandramohan^1^, Seth Owusu-Agyei^2^, Jayne Webster^1^, Frank Baiden^2^, Christopher Whitty^1^

^1^London School of Hygiene & Tropical Medicine, Keppel Street, London WC1E 7HT

^2^ Kintampo Health Research Centre, P.O.Box 200, Kintampo. Ghana, Tel/FAX +233 61 24145

**PI FOR CORRESPONDENCE, WITH E-MAIL AND MOBILE PHONE:**

Daniel Chandramohan

London School of Hygiene & Tropical Medicine, Keppel Street, London WC1E 7HT

Tele: + 44 (0) 207 927 2322, Email: daniel.chandramohan@lshtm.ac.uk

**LEAD PI(s) WITH E-MAIL AND MOBILE PHONE, WHERE DIFFERENT FROM CORRESPONDING PI*:**

**COLLABORATORS AND AFFILIATIONS:**

Jane Bruce^1^

^1^London School of Hygiene & Tropical Medicine, Keppel Street, London WC1E 7HT

**PROPOSED STARTING DATE: 1 January 2009**

**DURATION (MONTHS): 48 months**

**TOTAL BUDGET REQUESTED: US$ 1,473,510**

**TOTAL BUDGET SPEND IN NORTHERN INSTITUTIONS* US$**

**INSTITUTION WHERE GRANT TO BE HELD: London School of Hygiene & Tropical Medicine**

**SITE(S) OF RESEARCH (COUNTRIES, LOCATIONS, INSTITUTIONS):**

Kintampo Health Research Centre’s field research area in Ghana

**AIMS OF THE ACT CONSORTIUM THIS PROJECT ADDRESSES*- delete those what do not apply**

1. Improving drug targeting.

**Protocol version 2; date 1 December 2008**

**d. Stage 2 Component A: effects of restricted use of ACTs based on RDT results: a randomised controlled trial**

**d.1 Research questions** :

***Primary:***

What is the effect of restricting ACT to RDT positive cases of malaria compared to offering ACT on the basis of clinical judgement on the incidence of malaria in children < 4 years of age?

***Secondary:***

1. What is the effect of restricting ACT to RDT positive cases of malaria on the incidence of severe anaemia?
2. What is the effect of restricting ACT to RDT positive cases of malaria on the incidence of other severe febrile diseases?
3. What is the effect of RDT on the use of antibiotics for treating febrile illness?
4. Is restricting ACT to RDT positive cases of malaria cost-effective?
5. What is the effect of RDT on the community perception of the quality of care and the treatment seeking behaviour in particular for febrile illness in young children?

**d.2. Study design**: Cluster randomised two arm trial

**Study area**: The study will be conducted in Kintampo Health Research Centre field research area in Ghana. There are 36 government health facilities in the seven districts. The catchment population of these health facilities range from 10,000 to 85,000.

**d.3. Outcomes**

***Primary****:* Incidence of malaria (fever + any level of parasite density) in < 48 month-old children

***Secondary outcomes*:**

1. incidence of severe anaemia (Hb <8 g/dl) in < 48 month old children
2. incidence of any severe febrile illness in < 48 month old children
3. mean number of sick days in < 48 month old children
4. probability of using antibiotics for treating febrile illness in <48 month old children

**d.4. Study groups and enrolment of study children**:

The unit of randomisation will be health centres (HC). HCs will be randomly assigned either to the RDT+ACT group (ACT offered to RDT positive cases only) or to the clinical judgement+ACT group (ACT offered to all suspected cases of malaria by clinical judgement). Households within 10km radius of the HCs will be enumerated and one hundred <24 month old children from these households will be enrolled after obtaining informed consent from caretakers in each HC. At enrolment a photoID card of study children will be given to the caretakers and they will be advised to bring the photoID card when ever they bring the study children to the health facilities for treatment. The intervention and follow up will be continued for 24 months from enrolment.

**d..5. Inclusion/Exclusion criteria**

Children having chronic illnesses such as severe malnutrition and heart disease will be excluded from the study.

**d..6. Interventions**:

The study objectives will be explained to all staff involved in managing febrile cases in the study health centres (HCs). Staff in the RDT+ACT HCs will be trained in (1) using RDTs, (2) prescribing ACT to RDT positive cases only, and (3) further investigation of RDT negative cases for other infectious diseases. Staff in the clinical judgement + ACT HCs will undergo refresher courses in the clinical diagnosis of malaria and other infectious diseases with similar signs and symptoms, and management of malaria, and they will be advised to continue the current policy of offering ACT to all suspected cases of malaria. HC staff will identify study children using the PhotoID cards. Study children attending RDT+ACT HCs with a febrile illness will be tested with an RDT to confirm malaria and treated with ACT only if they have a positive test for malaria parasite. However if there are signs suggestive of other co-morbidities they will be treated with appropriate medicines in addition to AS+AQ. Children attending clinical judgement + ACT HCs with a febrile illness will be treated with ACT on the basis of the clinical judgement. Children and adults who are not enrolled in the study attending HCs in both arms with a febrile illness will be treated according to the current treatment guidelines ie.based on clinical judgement and using microscopy when feasible. The intervention will be continued for 24 months from the time of enrolment of study children.

**d.7. Measuring outcomes**:

***Incidence of malaria:***

Passive surveillance: All study children presenting with a history of fever to the study HCs will be identified using photoID cards and assessed with a morbidity questionnaire and a blood sample (blood slide and filter paper) will be collected to assess malaria parasite. Since the decision to offer an antimalarial drug will be based on RDT results or clinical judgment the blood slides will have limited use for treatment decision making. However, the blood slides will be at Kintampo Health Research Centre and the results will be reported to the health centres.

Active surveillance: Study children will be visited once a month at home and a morbidity questionnaire and blood samples (blood slide and filter paper) will be collected to assess malaria parasite if a child has a history of fever in the past 48 hours. Those who have a positive blood slide will be treated with ACT.

Both passive and active surveillance of malaria will be continued for 24 months from enrolment.

***Incidence of severe anaemia***:. All study children attending study HCs will be clinically assessed using a morbidity questionnaire and the Hb will be tested using a haemacue machine if anaemia is suspected by clinical examination. Children with Hb <11 gms/dl will be given appropriate treatment according to the national treatment protocol.

***Incidence of severe febrile illness and mean number of sick days:*** These outcomes will be measured in 100 episodes of febrile illness (50 in the wet and 50 in the dry season) in 10 randomly selected study HCs per arm. These children will be actively followed up on day 1,2,3 and 7 post treatment to assess the progress of their illness. If the illness has resolved by day 7, the number days the child was ill will be collected from the caretaker’s report. If the illness has not resolve by day 7 the child will be referred to the hospital and the number of days of illness will be collected from hospital discharge summary. The mean number of days of illness and the proportion of children requiring reference will be compared between the two groups.

***Use of antibiotics:*** The use of antibiotics will be monitored through the morbidity questionnaire completed during each visit to the study PHCs by study children.

**d.8. Sample size**:

***Effect of RDT+ACT on in the incidence of malaria***: To assess the effect of RDT+ACT on the incidence of malaria children under 48 months of age would require 30 clusters (15 per arm) and 200 PYAR per cluster on the basis of the following assumptions: (1) Incidence of malaria in the clinical judgement + ACT group would be 0.8 episode/ 1 PYAR (the rate observed in the IPTi group of <24 month old children in Navrongo, Ghana6); (2) The study would have 80% power at 95% significance level to detect a 20% difference in the incidence of malaria between the two comparison groups and this level of difference is deemed to be the non-inferiority margin of public health importance; (3) the coefficient of variation in the incidence of malaria between clusters will be 0.18

***Effect of RDT+ACT on in the incidence of severe anaemia***: Incidence of severe anaemia in the RDT + ACT group is expected to be 37/1000 PYAR (the rate observed in the 16-24 month old children who received placebo in Navrongo, Ghana6). The study would have 80% power at 95% significance level to detect a 36% reduction in the incidence of severe anaemia between the two comparison groups.

***Effect of RDT + ACT on febril children requiring referral:*** Actively following up 2000 episodes of febrile illness seen at 20 study HCs will have 85% power to detect 50% difference between the two arms assuming 1) risk of referral in the RDT + ACT group would 0.05; (2) the coefficient of variation in the risk of referral 0.1

Although mortality will be measured the study does not have adequate power to detect any difference between the groups.

**d..9 Effect of other malaria control interventions**

Coverage with other malaria control interventions, such as insecticide treated nets (ITNs) will be assessed during the prevalence surveys. Conclusions on the efficacy of the alternative case management strategies will be generalisable at the coverage levels of other interventions present in the study area. Antimalarial and other drugs are available in several formal and informal sector outlets and this may affect the adherence to the treatment regime of the study participants. Attempts will be made to maximise adherence to study protocol by participants through behaviour change communication strategies. At the monthly active follow up visits of study children the history of treatment received from any other health care provider other than the designated HCs will be documented and this information will be used to assess the extent of contamination of the study outcomes.

**d.10 Summary of data and biological sample collection per child**

| Procedures | enrolment | Unscheduled visits to PHCs | Monthly Scheduled home visits |
| --- | --- | --- | --- |
| Consent | √ |  |  |
| PhotoID | √ |  |  |
| Baseline socio-demographic, contextual factors CRF | √ |  |  |
| RDT testing |  | √ |  |
| ACT based on RDT result |  | √ (only In RDT+ACT PHCs) |  |
| Morbidity CRF |  | √ | √ (if history of fever in past 2 days) |
| Blood slide and filter paper sample |  | √ | √ (if history of fever in past 2 days) |
| Hb (Haemacue) |  | √ | √ (if history of fever in past 2 days) |

**d.11. Ethical issues**

Children in both arm of the study will be given standard care currently available in Ghana and all children will be closely monitored for any unexpected events. An informed written consent will be obtained from caretakers of the study children prior to enrolment. Ethical approval from the LSHTM and Kintampo Health Research Centre ethics committee will be obtained before starting the trial. The decision to progress to stage 2 of the study will be made by the Data Safety Monitoring Board.

**e. Stage 2 – component B: Cost effectiveness analysis**

**e.1.Research question**

**Primary**

What is the cost effectiveness of RDT based ACT for treatment of children under 4 years compared with ACT based on clinical judgement?

**Secondary**

What are the costs of alternative treatments where ACT is based on RDT results compared to ACT based on clinical judgement?

What are the cost implications for a District Health Management Team (DHMT) of making RDT based ACT policy?

What are the implications of the health insurance scheme for the cost effectiveness and equity of RDT based ACT?

**e.2. Study perspective**

The societal perspective will be taken and therefore will include costs to all those involved in the project including the children with fever and their carers. The provider perspective will be taken in assessing the cost implications for a DHMT of introducing RDTs into their health centres.

**e.3. Primary costs and outcomes**

**Costs**

Cost of treatment of febrile children with ACT based on positive RDT tests

Cost of treatment of febrile children with ACT based on clinical judgement

**Outcomes**

Incidence of malaria in children under 48 months of age

Incidence of sever anaemia in children under 48 months of age

Incidence of severe febrile illness in <48 month old children

**Secondary costs and outcomes**

**Costs**

Cost of treatment of febrile children under 48 months (ACTs + antibiotics + other treatments) in those tested with an RDT

Cost of treatment of febrile children under 48 months (ACTs + antibiotics + other treatments) based on clinical judgement alone

**Outcomes**

Mean number of sick days in children under 48 months of age

**e.4.Study site and population**

The study will link to Stage 2 – component A – “effects of restricted use of ACTs based on RDT results: a randomised controlled trial”**.** Outcomes will be measure in this RCT and costs will be measured in 7 out of the 28 health centres involved in the trial. The health centres involved in the costing exercise will be selected using simple random sampling from those selected for the trial.

**e.5.Costing study procedures**

Both financial and economic costs will be measured. The financial costs will include all direct expenditure on the trial including salaries, this will include stage 2 components only, the costs of stage 1 components will not be included. The economic costs will include financial costs plus any opportunity costs including travel time to health facilities. Capital costs will include any equipment purchased for the trial that will be used for more than one year. Development and set up costs involving training of health centre staff will be included as capital costs as their impact is expected to be for more than one year. A costing framework will be used to categorise all costs and their categories and will include 1) design development, 2) project set-up, and 3) project implementation. Findings from the delivery systems determinants study, Stage 1 component B will be used to identify cost categories.

**e.6.Data analysis**

Both cost effectiveness ratios and an incremental cost and consequences analysis of RDT based versus clinical judgement based ACT will be assessed. Modelling of the outcomes to Disability Adjusted Life Years (DALYs) will be undertaken as per ACT Consortium economic evaluation guidance.

**e.7. Ethics**

Ethics permissions will be requested jointly with the RCT Stage 2 – component A.

**f. Timetable**

| Activities | Q1 | Q2 | Q3 | Q4 | Q5 | Q6 | Q7 | Q8 | Q9 | Q10 | Q11 | Q12 | Q13 | Q14 | Q15 | Q16 |
| --- | --- | --- | --- | --- | --- | --- | --- | --- | --- | --- | --- | --- | --- | --- | --- | --- |
| Project preparation (staff recruitment & logistics) | √ |  |  | √ |  |  |  |  |  |  |  |  |  |  |  |  |
| Sensitisation of community & health system | √ |  |  | √ |  |  |  |  |  |  |  |  |  |  |  |  |
| Staff training, development and field testing of tools | √ |  |  | √ |  |  |  |  |  |  |  |  |  |  |  |  |
| Enrolment and follow up of participants for the accuracy of RDT and treatment outcome study |  | √ | √ | √ |  |  |  |  |  |  |  |  |  |  |  |  |
| Round 1: health facility observations, staff interviews and exit interviews with carers |  | √ |  |  |  |  |  |  |  |  |  |  |  |  |  |  |
| Round 2: health facility observations, staff interviews and exit interviews with carers |  |  | √ |  |  |  |  |  |  |  |  |  |  |  |  |  |
| Training of health centre staff |  |  |  |  | √ |  |  |  |  |  |  |  |  |  |  |  |
| Enumeration & enrolment of study children for the randomised trial |  |  |  |  | √ |  |  |  |  |  |  |  |  |  |  |  |
| Implementation of intervention |  |  |  |  | √ | √ | √ | √ | √ | √ | √ | √ | √ | √ |  |  |
| Active and passive surveillance of outcomes |  |  |  |  | √ | √ | √ | √ | √ | √ | √ | √ | √ | √ |  |  |
| Programme costs for cost effectiveness study |  |  |  | √ | √ | √ | √ | √ | √ | √ | √ | √ | √ | √ |  |  |
| Health facility surveys and exit interviews for cost effectiveness analysis |  |  |  |  |  |  |  |  |  |  |  | √ |  |  |  |  |
| Data management |  | √ | √ |  | √ | √ | √ | √ | √ | √ | √ | √ | √ | √ | √ |  |
| Analysis and dissemination |  |  |  | √ |  |  |  |  |  |  |  |  |  |  | √ | √ |

**References**

Reyburn H, Mbatia R, Drakeley C et al. Overdiagnosis of malaria in patients with severe febrile illness in Tanzania: a prospective study. BMJ. 2004;329:1212-5.

^2^ World Health organisation. 2003. Malaria Rapid Diagnosis, Making it Work.Rs/2003/GE/05(PHL)

^3^ Phillips-Howard PA, Wannemuehler KA, ter Kuile F et al. Diagnostic and prescribing practices in peripheral health facilities in rural western Kenya. Am-J-Top-Med Hyg. 2003; 68:44-9.

^4^ Cruciani M, Nardi S, Malena M, Bosco O, Serpelloni G, Mengoli C. Systematic review of accuracy of the ParaSight-F test in the diagnosis of plasmodium falciparum malaria. Medical Science Monitor 2004; 10:81-8.

^5^ Swarthout TD. Counihan H. Senga RK. van den Broek I. Paracheck-Pf accuracy and recently treated Plasmodium falciparum infections: is there a risk of over-diagnosis?. Malaria Journal.2007; 6:58, 2007.

^6^ Guthmann JP. Ruiz A. Priotto G. Kiguli J. Bonte L. Legros D. Validity, reliability and ease of use in the field of five rapid tests for the diagnosis of Plasmodium falciparum malaria in Uganda**.** *Transactions of the Royal Society of Tropical Medicine & Hygiene 2002; 96:254-7, 2002.*

^7^ Singh N. Saxena A. Usefulness of a rapid on-site Plasmodium falciparum diagnosis (Paracheck PF) in forest migrants and among the indigenous population at the site of their occupational activities in central India**.** *American Journal of Tropical Medicine & Hygiene 2005;. 72:26-9.*

^8^ Chandramohan D, Owusu-Agyei S, Carneiro I et al. Cluster randomised trial of intermittent preventive treatment for malaria in infants in an ares of high, seasonal transmission in Ghana. BMJ 2005; 331: 727-733.

^9^ Cisse B, Sokhna C, Boulanger et al. Seasonal intermittent preventive treatment with artesunate and sulfadoxine-pyrimethamine prevents malaria in Senegalese children Lancet 2006; 367:659-667.

^10^ Schellenberg D, Menendez C, Kahigwa E et al. Intermittent treatment for malaria and anaemia control at time of routine vaccinations in Tanzanian infants: a randomised,placebo-controlled trial Lancet 2001; 357: 1471-77.

^11^ Sources and prices of selected products for the prevention, diagnosis and treatment of malaria. WHO, RBM, UNICEF, UNAIDS, PSI, MSH 2004.
